# Application of Machine-Learning Algorithms for Better Understanding of Tableting Properties of Lactose Co-Processed with Lipid Excipients

**DOI:** 10.3390/pharmaceutics13050663

**Published:** 2021-05-05

**Authors:** Jelena Djuris, Slobodanka Cirin-Varadjan, Ivana Aleksic, Mihal Djuris, Sandra Cvijic, Svetlana Ibric

**Affiliations:** 1 Department of Pharmaceutical Technology and Cosmetology, University of Belgrade-Faculty of Pharmacy, Vojvode Stepe 450, 11221 Belgrade, Serbia; ivana.aleksic@pharmacy.bg.ac.rs (I.A.); sandra.cvijic@pharmacy.bg.ac.rs (S.C.); svetlana.ibric@pharmacy.bg.ac.rs (S.I.); 2 Hemofarm STADA A.D., Beogradski put bb, 26300 Vršac, Serbia; slobodanka.cirin-varadjan@hemofarm.com; 3 Department of Catalysis and Chemical Engineering, Institute of Chemistry, Technology and Metallurgy—National Institute of the Republic of Serbia, University of Belgrade, Njegoševa 12, 11000 Belgrade, Serbia; mihal.djuris@ihtm.bg.ac.rs

**Keywords:** co-processed excipients, compaction analysis, machine learning, neural networks, multilayer perceptron, sensitivity analysis, tensile strength, lipid excipients, lactose, monohydrate

## Abstract

Co-processing (CP) provides superior properties to excipients and has become a reliable option to facilitated formulation and manufacturing of variety of solid dosage forms. Development of directly compressible formulations with high doses of poorly flowing/compressible active pharmaceutical ingredients, such as paracetamol, remains a great challenge for the pharmaceutical industry due to the lack of understanding of the interplay between the formulation properties, process of compaction, and stages of tablets’ detachment and ejection. The aim of this study was to analyze the influence of the compression load, excipients’ co-processing and the addition of paracetamol on the obtained tablets’ tensile strength and the specific parameters of the tableting process, such as (net) compression work, elastic recovery, detachment, and ejection work, as well as the ejection force. Two types of neural networks were used to analyze the data: classification (Kohonen network) and regression networks (multilayer perceptron and radial basis function), to build prediction models and identify the variables that are predominantly affecting the tableting process and the obtained tablets’ tensile strength. It has been demonstrated that sophisticated data-mining methods are necessary to interpret complex phenomena regarding the effect of co-processing on tableting properties of directly compressible excipients.

## 1. Introduction

Increase in the new forms of active pharmaceutical ingredients (APIs), in terms of their physicochemical and stability profiles, has put a great pressure to the selection of excipients with the appropriate functionalities. In terms of large-scale manufacturing, co-processing (CP) of excipients has become a reliable option to facilitated formulation and manufacturing of variety of solid dosage forms for oral administration. CP provides superior properties to excipients, compared to the simple physical mixture of the components, and yet the absence of chemical changes ensures the safety of the novel excipients. The worldwide market of co-processed excipients (CPEs) is steadily growing and is expected to exceed USD 2.6 billion in 2027 [1].

Development of directly compressible formulations with high doses of poorly flowing/compressible API remains a great challenge for the pharmaceutical industry [2]. Franc et al. have reviewed benefits of CPEs for direct compression of tablets, especially in terms of their positive influence on API content uniformity and tablets’ mass variation, disintegration, dissolution, mechanical resistance and stability [3]. Other studies report that CPEs had improved formulations’ tableting properties [4,5,6]. Although spray-drying is still the predominant method for development of CPEs, wet-granulation, melt-granulation and melt-extrusion have also gained attention [1,7,8,9]. Selection of the binder used for the co-processing can be a predominant factor that affects tableting properties of CPEs [10].

Dynamic compaction analysis has been recognized as a valuable tool for evaluation of directly compressible CPEs and development of high-load tablets of poorly flowable/compressible API [2,11]. Osamura et al. [12] have demonstrated how small-scale measurements of tableting properties can aid formulation development and assessment of excipients’ functionality. Direct compression is, in essence, a relatively simple process whereby the powder mixture is compressed by applying a sufficient compaction pressure. Once the compaction phase is over, tablet needs to be detached from the tableting punch and ejected from the die. Most problems, in the industrial manufacturing of tablets, come from the lack of understanding of the interplay between the formulation properties, process of compaction, and stages of tablets’ detachment and ejection. E.g., it has been demonstrated recently that detachment process affects tablets’ tensile strength [13]. Excessive lubrication is often needed to overcome tablets’ sticking, lamination, and capping. Furthermore, wearing of tableting tools, as a result of inappropriate compaction pressures, frequently occurs [14]. Due to the complex nature of the interaction between the formulation, compaction process, and the obtained tablets’ mechanical properties, it seems that sophisticated data-mining methods, such as machine-learning (ML) tools, are required for the appropriate interpretation of such phenomena. In opposition to the traditional statistical methods, ML tools offer a distinct opportunity to model complex relationships between several input and output data, thus gaining valuable insights on the process of interest and allowing accurate predictions. Historically, ML tools, such as artificial neural networks (ANN), have been predominantly used to optimize formulation composition and/or processing parameters, based on product properties that are routinely assessed (e.g., drug release profile, tablet disintegration time, hardness and friability) as indicators of a formulation performance [15,16,17,18,19,20,21]. In addition, some review papers also highlight various applications of ML methods in the development of solid dosage forms [22,23,24,25,26]. One of the first mentions of ML tools applied to material tableting properties, and the related compression/compaction phenomena, is the study of Bourquin et al. [27] who used ANN to quantitatively assess the influence of excipients concentration on the ejection and residual forces during tablet ejection. Due to the non-linear relationship between the investigated input variables and the resulting ejection properties, they found that ANN performs much better than classical response surface methodology (RSM) modeling technique. The study of Belic et al. [28] highlighted that ANN and fuzzy models can be used for interpretation of the material particle size and tableting machine settings effect on the resulting tablet capping tendency. Khalid et al. [29] used different machine-learning tools (decision trees, random forests, fuzzy systems, ANN, symbolic regression) to model the dependence of tablet tensile strength on the formulation (varying excipients quantities) and process parameters (varying compaction machines, pressures and speeds), highlighting the importance of predicting tensile strength as an important indicator of tablets mechanical properties. Lou et al. [30] used six different machine-learning algorithms to model the relationships between the raw material type (core/shell powders vs. physical mixtures) and ingredients concentration, as well as the materials compactibility (measured as tensile strength and brittleness index). According to their results, all the applied algorithms provided acceptable predictability and indicated improved compactibility of core/shell powders. Similarly, Millen et al. [31] assessed the effect of the selected formulation and process variables (categorical and numerical) on the compressibility, compactibility, and manufacturability of a granulate blend. A recent study of Khalid and Usman [32] confirmed the utility of ML models for characterization of pharmaceutical excipients.

Lactose is one of the most commonly used diluents for tablets and there are already numerous examples of novel CPEs that have been developed to provide multifunctionality and/or enable direct compression [3,33]. However, none of these CPEs contains conventional lubricating aids, such as magnesium-stearate, due to its insolubility in water and difficult processing via the common CP methods, such as spray-drying. Therefore, CPE usually require additional lubricating aids. Compritol^®^ (glyceryl behenate) and Precirol^®^ (glyceryl palmitostearate) are well-known lipophilic glycerides with low HLB (hydrophilic-lipophilic balance) values that can be used as lubricants or matrix-forming agents in the conventional solid oral dosage forms [34] and are also suitable for several melting-based processing methods [35]. Melt-granulation has already been recognized as a suitable CP method for directly compressible CPEs [9].

The aim of this study was to analyze the influence of the compression force (load), co-processing of excipients, and the addition of the model API (paracetamol) on the obtained tablets’ tensile strength and the specific parameters of the tableting process, such as (net) compression work, elastic recovery, detachment, and ejection work, as well as the ejection force. Lactose monohydrate was co-processed with Compritol^®^ or Precirol^®^ by the melt-granulation method. Due to the multivariate nature of the studied parameters, and the inclusion of a categorical variable (physical mixtures vs. co-processed excipients), neural networks were used to build prediction models and identify the variables that are predominantly affecting the tableting process and the obtained tablets’ tensile strength. To the best of our knowledge this is the first study that uses ML algorithms for interpretation of the effect of co-processing on the tableting properties.

## 2. Materials and Methods

### 2.1. Materials

Paracetamol (Acros Organics, Geel, Belgium) was used as the model active pharmaceutical ingredient (API). Lactose monohydrate (Carlo Erba Reagents, Milan, Italy) was used as a diluent. Precirol^®^ ATO 5 (glyceryl palmitostearate) and Compritol^®^ 888 ATO (glyceryl behenate) kindly supplied by Gattefossé S.A.S. (Saint-Priest, France) were used as the lipid meltable binders.

### 2.2. Co-Processing of Excipients

In situ fluid bed melt-granulation process with Precirol^®^ was performed in Mycrolab bed processor (OYSTAR Hüttlin, Schopfheim, Germany) while granules with Compritol^®^ were obtained in a custom—made fluid bed. The batch size was 200 g. To obtain the novel co-processed lactose-based excipients, different amount of the Precirol^®^ or Compritol^®^ (10 or 15% (*w*/*w*)) were used. All starting materials, lactose monohydrate (90 or 85% (*w*/*w*)) and lyophilic, meltable binders Precirol^®^ or Compritol^®^ were filled in the fluid bed processor, fluidized and preheated to the product temperature of 65 °C (Precirol^®^) or 85 °C (Compritol^®^). The starting point of granulation was defined as the time when product temperature reached the required temperature. The inlet air heating was switched off 10 min after the products reached predefined temperature. During the whole process, the inlet air flow rate was kept at the approximately 30 m^3^/h. The final point and the end of the fluidization process was when the products temperature decreased below 30 °C. The fluid bed processor was stopped, and the obtained granules were collected.

### 2.3. Dynamic Compaction Analysis

The comprehensive dynamic compaction analysis of the investigated excipients was conducted using an instrumented laboratory Gamlen tablet press (GTP D series, Gamlen Tableting Ltd., Nottingham, UK). The compaction studies were performed using the single punch tablet press with cylindrical flat-faced punches (6 mm diameter). In this study, compacts (100 mg) were compressed under the compression loads of 100 kg, 250 kg and 500 kg and at the compaction speed of 60 mm/min. Immediately after compression, the resistance to crushing of tablets was measured. The measuring of the resistance to crushing (Erweka tablet hardness tester TBH125D, Langen, Germany) was performed on at least three samples. Based on the obtained values of the resistance to crushing, the tensile strength (σ) of the investigated samples was calculated using the following equation:

σ = 2F/πDt(1)

where F is the applied force for tablet breaking, D is the compact diameter and t is the out-of-die compact thickness.

Total work of compression (sum of net compression and elastic work), detachment work and ejection work were calculated from force-displacement curves that have been generated using the supporting software of the tablet press. Based on the constructed displacement profile, the compression, detachment, and ejection profiles were determined. The area under the compression force-displacement curve is the total work of compression and using the trapezoidal method both the total and the net compression work can be calculated. Detachment and ejection force-displacement curves were generated by the instrument software as well and represent the work used during detachment and ejection phase, respectively. Ejection force has been evaluated based on the recorded maximum value of the force during the ejection phase.

Percentage of in-die elastic recovery was determined, based on the difference between the upper punch base position and punch positions corresponding to maximum and minimum values of compression load, using the following equation:

Elastic recovery (%) = (Hmax − Hmin)/Hmin × 100%,(2)

where Hmax and Hmin represent the in-die compact thickness corresponding to maximum and minimum values of the compression load, respectively.

**Figure 1:**
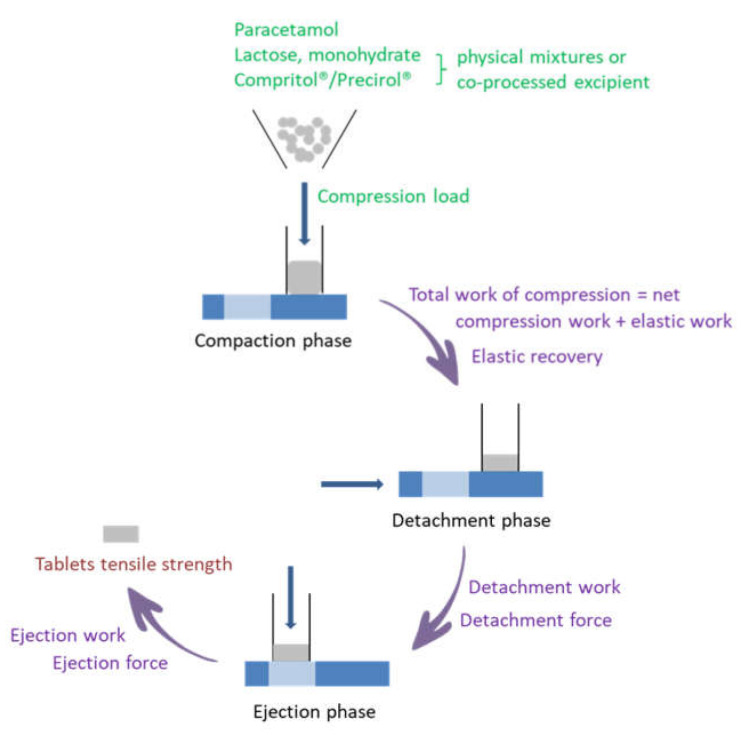
Schematic representation of the dynamic compaction analysis and studied parameters.

As represented in Figure 1, during the dynamic compaction analysis several process parameters can be obtained. In the present study, process parameters highly correlated process parameters have been excluded from further analysis: elastic work (since it is calculated as the difference between TWC (total work of compression) and NWC (net work of compression)) as well as detachment force due to high correlation with DW (detachment work).

### 2.4. Artificial Neural Networks

Statistica version 13.5.0.17 (TIBCO Software Inc., Frankfurt am Main, Germany) was used to build and analyze different neural networks to investigate the influence of the input parameters (co-processing with the lipid excipients (binders), content of the lipid excipients, addition of API, compression load) on the seven outputs. The outputs will be hereinafter referred to as TS (tensile strength), TWC (total work of compression), NWC (net work of compression), ER (elastic recovery), DW (detachment work), EJW (ejection work) and EF (ejection force). Two types of neural networks were used to analyze the data: classification (Kohonen network, KN) and regression networks (multilayer perceptron, MLP, and radial basis function, RBF). Kohonen network was used to identify clusters in the data. Regression neural networks were developed to model the aforementioned outputs both separately and in combination.

Dataset for neural networks development and analysis consisted of the 90 entries that covered variation in the compression load (100 and 500 kg), content of Compritol^®^ or Precirol^®^ in the physical mixtures or co-processed excipients (0, 10 and 15%) and the content of API (0, 25 and 50%), according to Table 1. Each formulation was tested in triplicate. Apart from the numerical variables, the state of excipients was introduced as a categorical variable with the two entries: physical mixture (PM) and co-processed (CS) excipient. This dataset was split into training (70% of the data), test (15% of the data) and validation (15% of the data). Additional dataset was used for external validation of the developed neural network models, and it contained information on the 8 formulations that were made by varying the content of API (25 and 50%) and using Compritol^®^ or Precirol^®^ in the form of PM or CS excipients for the formulation. Compression load of 250 kg was used for tableting during the preparation of the external validation dataset formulations. Summary of the data, i.e., formulations’ properties and compression load that were used in the study, is represented in Table 1.

In Statistica software, Kohonen neural network was first built. Kohonen training algorithm was used in automated neural networks cluster analysis, with the training, testing, and validation data (Table 1) used to construct the network. All variables were selected (formulation composition, compression load, co-processing of excipients, as well as the obtained tablets’ TS, TWC, NWC, ER, DW, EJW, and EF). Dimensions of the topological map were set to 2 × 2 (topological height × width) of the self-organizing feature map (SOFM) and the training cycles were set to 1000. Upon training of the network, the obtained clusters and network’s weights were assessed, and the external validation data was also analyzed in terms of identification of the cluster that the data is mostly similar to.

Afterwards, automated search for regression neural networks (MLP or RBF) was performed. A total of 20 networks, either MLP or RBF, were trained and 5 of them were to be retained, according to the training, test, and validation performance. The following activation functions were tested for both hidden and output neurons: identity, logistic, Tanh, and exponential. Broyden–Fletcher–Goldfarb–Shanno (BFGS) was used as a powerful second order training algorithm. Although conjugate gradient algorithms are more often used due to the quicker calculations, BFGS generally converges in fewer iterations even though it requires more computations [36]. Radial basis function was used as the training algorithm. Gaussian and identity functions were used as activation functions for hidden and output neurons, respectively. For both MLP and RBF networks, sum of squares (SOS) was used as the error function.

To assess the performance of the developed neural network models, Pearson correlation coefficient was calculated between the experimentally obtained and values predicted by neural networks. Global sensitivity analysis was performed to evaluate the relative importance of the input variables for the developed neural network models. According to the supporting documentation [37] Statistica calculates the ratio of the network error with a given input omitted to the network error with the input available, to determine how sensitive the model is to that input. If the ratio is 1 or less, that input variable can be pruned from the network.

**Figure 2:**
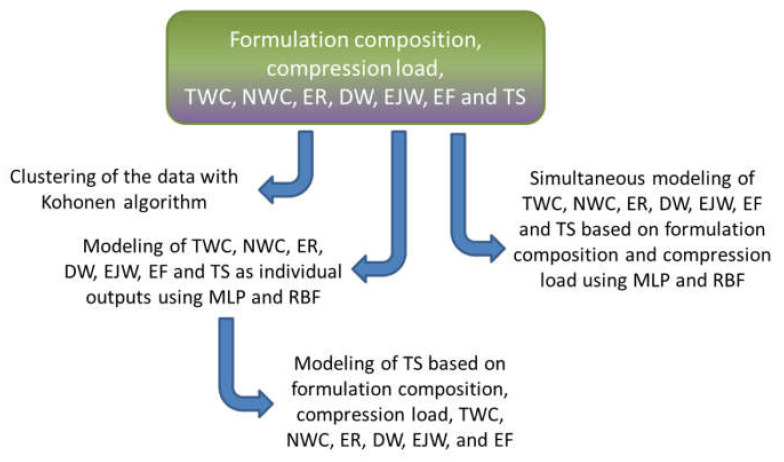
Schematic representation of the workflow of machine-learning algorithms that were applied for data analysis in the presented study (abbreviations: DW—detachment work, EF—ejection force, EJW—ejection work, ER—elastic recovery, MLP—multilayer perceptron, NWC—net work of compression, RBF—radial basis function, TS—tensile strength, TWC—total work of compression).

Figure 2 represents the workflow of machine-learning algorithms and data analysis that was performed in this study.

## 3. Results and Discussion

### 3.1. Clustering with Kohonen Neural Networks

**Figure 3:**
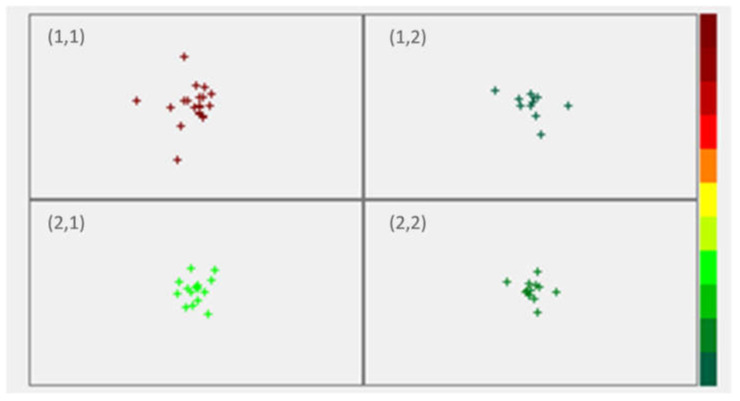
Self-organizing feature map for clustering of the training data. Positions are occupied by physical mixtures (1,1) and co-processed excipients (1,2) compressed at 500 kg, followed by physical mixtures (2,1) and co-processed excipients (2,2) that compressed at 100 kg.

Kohonen neural networks were applied as a clustering algorithm to search for the similarities in the data. Kosugi et al. [13] have recently demonstrated that self-organizing maps can be successfully used for classification of powders with different properties in distinct clusters. Our aim was to assess whether a classification algorithm could identify patterns in the available data, including both inputs and outputs. Once the Kohonen algorithm was applied with the pre-set 2 × 2 architecture, 2 × 2 SOFM (represented in Figure 3) was obtained.

**Figure 4:**
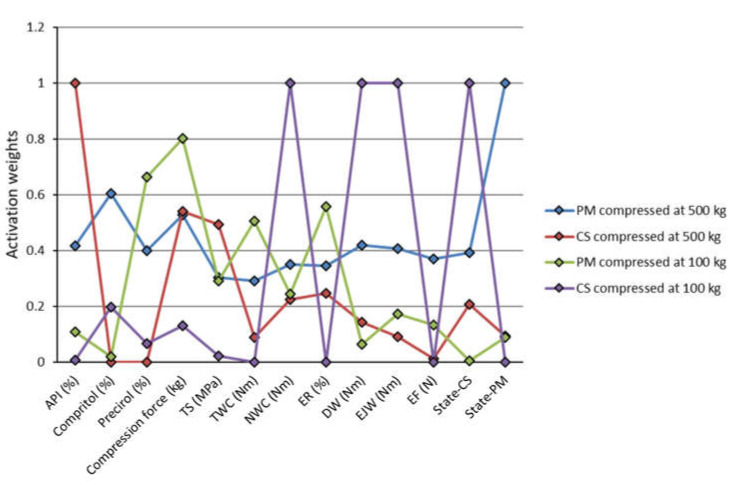
SOFM weights for all variables (abbreviations: API—active pharmaceutical ingredient, CS—co-processed, DW—detachment work, EF—ejection force, EJW—ejection work, ER—elastic recovery, NWC—net work of compression, PM—physical mixture, TS—tensile strength, TWC—total work of compression).

As represented in Figure 3, and according to the obtained activations, developed SOFM 13-4 network has clustered all data in the following manner: position (1,1) is occupied by physical mixtures compressed at 500 kg, position (1,2) refers to co-processed excipients compressed also at 500 kg, followed by positions (2,1) and (2,2) that are occupied by physical mixtures and co-processed excipients, respectively, compressed at 100 kg. Therefore, Kohonen neural network has recognized co-processing and the compression load as the predominant factors that affect variability in the data. The obtained networks’ weights for all variables are represented in Figure 4. SOFM has separated all samples appropriately into the four listed clusters, and the obtained weights provide means to identify variables that separate the clusters. Differences in networks’ weights may be indicative of the variables that are affecting properties of specific formulations. As such, model can be used to classify additional samples.

As described in the Methods section, external validation data (Table 1) was used for classification and the obtained results are represented in Table 2.

Formulations presented in Table 2 were all made at compression load of 250 kg. SOFM has clustered all PM formulations (regardless of the API and lipid binder content) in the position (2,1) that corresponds to the training data of PM formulations compressed at 100 kg load. Similarly, all CS formulations were grouped within the (2,2) position that corresponds to the training data of CS formulations compressed at 100 kg. Therefore, the obtained model has successfully separated PM from CS formulations, and has classified samples compressed at 250 kg compression load as being similar to clusters of 100 kg compression load samples.

### 3.2. Modeling of the Individual Outputs

As described in the Methods section, the next step was development of regression models, based on MLP and RBF networks, to model the influence of the input variables on each of the studied outputs. Once the data were input, according to the software specific settings MLP networks were selected with the minimum of 3 and a maximum of 11 hidden units, whereas RBF networks were selected with the minimum of 13 and maximum of 18 hidden units. Networks were built and the obtained results are represented in Table 3. Table 3 reports the optimal MLP and RBF networks that were selected based on their performance during the network development (training, test and validation data) and additional external validation.

Names of neural networks represent the number of neurons in the input, hidden, and the output layer. Therefore, in Table 3 each MLP network had one output that was predicted based on five inputs (a bias term, the amount of API, Compritol^®^ and Precirol^®^; compression load and state of the excipients) and varying number of neurons in the hidden layer. The number next to the BFGS training algorithm refers the number of cycles that were necessary for networks to reach the optimal outcomes. Automated search was used to facilitate the selection of the activation functions that are also represented in Table 3. Correlation coefficient for the external validation data was used as a main criterion to evaluate the overall networks’ performance. The obtained data illustrate that MLP and RBF networks were, in general, able to identify and model the influence of the input variables on the target outputs. Predictions for TS of the external validation tablets were the least accurate. Since this was in fact the most important of the target outputs, additional efforts were put to build new models for TS prediction and are represented in the subsequent sections.

**Figure 5:**
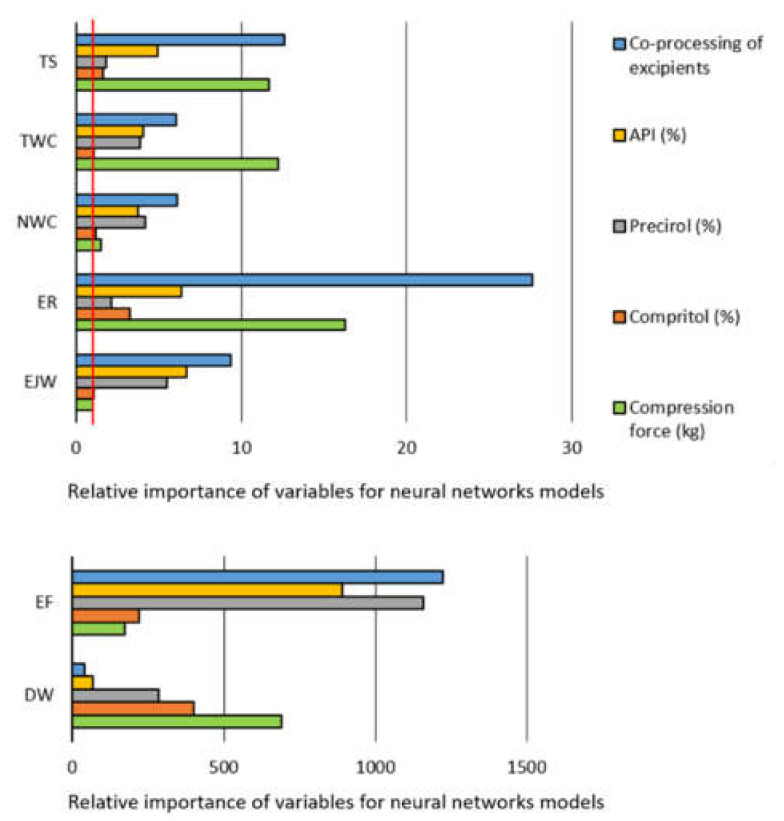
Sensitivity analysis for neural networks models that successfully predict tableting process properties based on the formulation composition, state of the excipients, and the compression load (abbreviations: DW—detachment work, EF—ejection force, EJW—ejection work, ER—elastic recovery, NWC—net work of compression, TS—tensile strength, TWC—total work of compression). Red line in the upper subfigure denotes the sensitivity margin (value of 1).

Sensitivity analysis was used to identify the input variables that are especially important for the developed models, i.e., for successful prediction of the target output variables. Sensitivity analysis was performed for RBF model for TS, and for the MLP models for all other target outputs excluding TS. Selection of neural network models for the sensitivity analysis was based on the highest performance, i.e., overall correlation coefficient. The obtained results are represented in Figure 5. Two graphs were used due to the differences in the size ranges of the obtained values.

Based on the represented sensitivity analysis, it can be concluded that apart from the compression load, which was expected to affect the tested materials’ tableting properties [38], formulations’ composition (API, Precirol^®^ and Compritol^®^ content) and the excipients co-processing were also identified as significant. The margin for the significant model terms is marked by red vertical line in Figure 5. In the case of EF and DW all terms substantially exceed the sensitivity margin.

**Figure 6:**
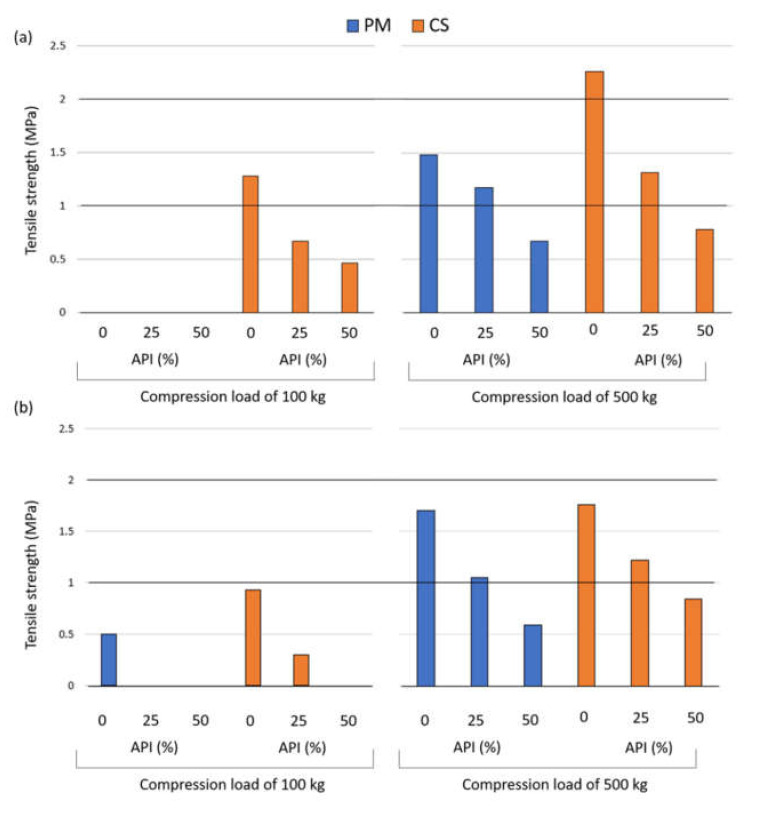
Influence of the compression load (kg), co-processing and the amount of API (%) on the TS of tablets formulated with (**a**) Compritol^®^ or (**b**) Precirol^®^. Abbreviations: API—active pharmaceutical ingredient, CS—co-processed, PM—physical mixture.

TS model was affected to the same extent, approximately, by the co-processing and the compression load. The influence of the amount of API and co-processing on TS of tablets prepared at 100 and 500 kg compression loads is represented in Figure 6. As for the compression at 100 kg, the highest TS was obtained for the co-processed excipient with Compritol^®^ (1.28 MPa), followed by the co-processed excipient with Precirol^®^ (0.93 MPa). The inability to form compact/tablet of tensile strength sufficient for measurement is represented by missing bars in Figure 6. It is also important to mention that pure lactose monohydrate was not compactible at 100 kg compression load, whereas the lactose compact prepared at 500 kg had TS of 1.28 MPa.

**Figure 7:**
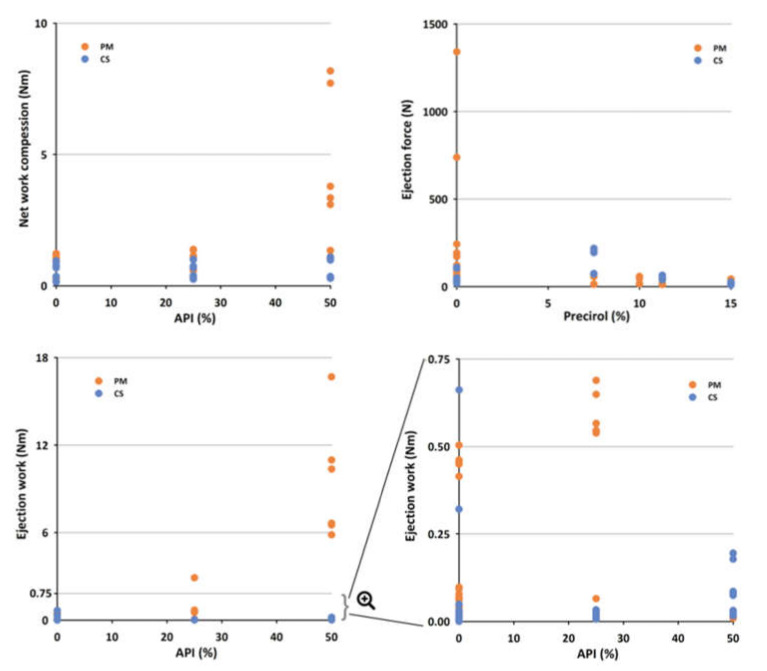
The influence of co-processing on NWC, EF, and EJW for formulations containing varying amounts of API. Abbreviations: API—active pharmaceutical ingredient, CS—co-processed, EF—ejection force, EJW—ejection work, NWC—net work of compression, PM—physical mixture.

Neural network model for TWC was predominantly affected by the compression load, whereas in the case of NWC and ER co-processing had the major effect. With the increase in compression load TWC increased as well, as expected. In the case of excipients co-processed with both Compritol^®^ and Precirol^®^, NWC was lower compared to the physical mixtures, especially for formulations that contained 50% of API (Figure 7). It is important to emphasize that co-processing has led to the increase in TS and decrease in NWC at the same time. This means that the energy generated during the process of compression was more efficiently exploited. Changes in NWC are indicative of changes in the deformation mechanism, and as such may be useful for process development and control [39].

Sensitivity analysis has further revealed that the model for DW is mostly affected by the compression load, followed by the amount of the lipid binders, whereas co-processing of excipients, amount of API (%) and Precirol^®^ impact models for EJW and EF (Figure 5). Co-processing leads to the lower EJW values, whereas EJW increases with the increase in API (%). It is important to emphasize that co-processing also reduced EF, with the smallest forces being required to eject tablets containing high amounts of Precirol^®^ (Figure 7). EF and/or EJW are much more often reported and discussed compared to their detachment counterparts. The obtained findings illustrate that it may lead to neglecting of the compression load effect on DW. DW often needs to be decreased, to avoid capping and/or lamination tableting issues, and it can be achieved by lowering the compression load and/or addition of lubricants [40].

### 3.3. Modeling of Tablets Tensile Strength

**Figure 8:**
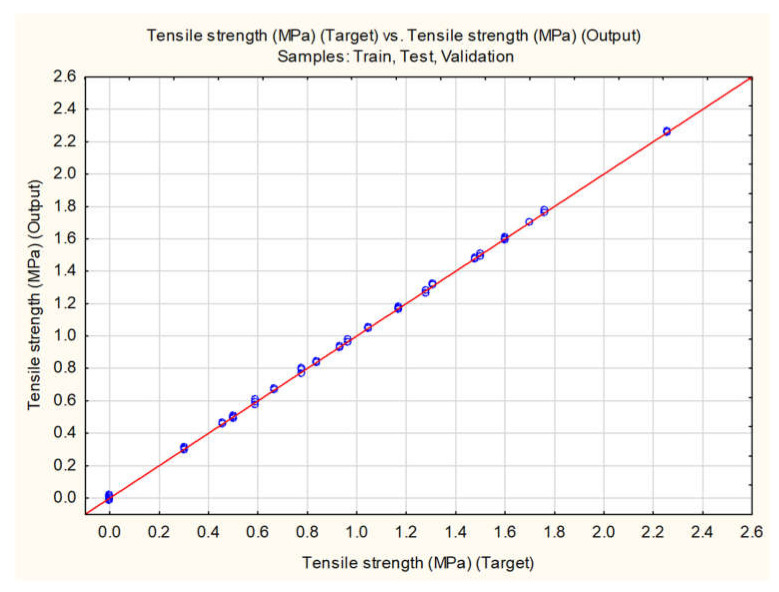
Correlation between the experimentally obtained and values for the tensile strength predicted by MLP (multilayer perceptron) 12-11-1 neural network.

As accentuated previously, tablets’ TS is an essential parameter for characterization since it is representative of tablets’ mechanical properties. During the previously presented modeling approaches in Section 3.2, it was evident that the models’ ability to predict tablets’ TS based on the available inputs can be improved. Therefore, MLP networks were further evaluated, in terms of changing the status of TWC, NWC, ER, DW, EJW, and EF to inputs, and analyzing TS as the sole output of the network. The automated search was performed, as previously described, and MLP 12-11-1 network was the obtained network selected for evaluation due to its high predictive abilities, as represented in the Table 4 and Figure 8.

For the MLP 12-11-1 network, correlation coefficient between the experimentally obtained and values predicted for the external validation dataset was 0.9263. To compare the prediction ability of the model with the previously reported ML-based models [29], normalized RMSE (root mean squared error) of 11.72% was calculated. Performance of MLP 12-11-1 is comparable to the published results [29], in terms of error metric, whereas the correlation coefficient is higher in the present study.

This has proved that apart from the co-processing, formulation composition, and the compression load, parameters monitored during different stages of tableting can also be correlated with tablets’ TS. Global sensitivity analysis has revealed that EF and EJW are, among the additional inputs, those with the highest impact to the model for prediction of TS.

The same search was also performed with RBF networks and the optimal RBF network provided correlation coefficient of 0.8738 and higher errors in prediction, for the external dataset, making MLP 12-11-1 favorable network for TS prediction.

### 3.4. Development of Models for Simultaneous Predictions of Seven Outputs

In the next step MLP and RBF networks were trained to model seven outputs (TS, TWC, NWC, ER, DW, EJW, and EF) simultaneously, i.e., models were developed to predict seven outputs at the same time. As described previously, automated network search was performed and details on the developed networks are represented in Table 5. Five best performing artificial neural networks (three of them were MLP and two were RBF networks) were selected for further evaluation.

**Figure 9:**
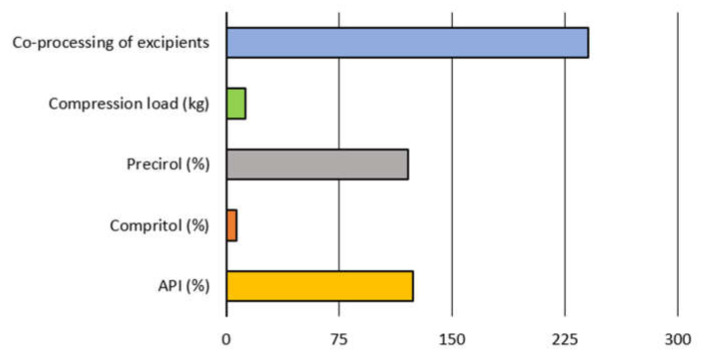
Sensitivity analysis for MLP model developed to successfully predict seven outputs simultaneously (TS, TWC, NWC, ER, DW, EJW, EF), based on the formulation composition, state of the excipients, and the compression load. Abbreviations: API—active pharmaceutical ingredient, DW—detachment work, EF—ejection force, EJW—ejection work, ER—elastic recovery, MLP—multilayer perceptron, NWC—net work of compression, TS—tensile strength, TWC—total work of compression.

Based on the obtained results MLP 6-11-7 network, with BFGS 215 training algorithm, was selected for further analysis, due to its highest overall performance. This network consists of six neurons in the input layer, 11 neurons in the hidden layer and seven outputs. Activation functions for its hidden and output layer were exponential and Tanh, respectively. Sensitivity analysis performed for MLP 6-11-7 network revealed that the model is, overall, mostly affected by the co-processing of excipients, amount of API (%) and Precirol^®^ (%), as represented in Figure 9. All studied input parameters exceed the models’ sensitivity margin.

## 4. Conclusions

The presented results support further development and usage of CPEs obtained by the melt-granulation procedure with the lipophilic glycerides. It has been demonstrated that CPE have superior properties compared to their physical mixture counterparts. Developed CPE enable direct compression without the necessity for additional lubricating aids. Furthermore, glycerides have already been recognized for their potential to be used as matrix-forming excipients [41], therefore these CPEs could be used for direct compression of matrix tablets with modified drug release.

This study has confirmed the great potential of ML algorithms for the assessment of direct compression process. For the first time, different neural networks were applied to study the influence of co-processing, compression load, and formulation composition on the obtained tablets’ TS, TWC, NWC, ER, DW, EJW, and EF. It has been demonstrated that co-processing has affected positively tablets’ TS and facilitated the process of direct compression. Classification neural network, based on Kohonen algorithm, was designed and tested. It has efficiently clustered samples, based on the co-processing and the compression load. Both types of regression neural networks that were tested, MLP and RBF, have been demonstrated as suitable for the sensitivity analysis. MLP networks have slightly outperformed, especially in terms of an MLP model that was developed for the successful prediction of tablet’ TS with high correlation coefficient and acceptable prediction error. Validation of both classification and regression models was demonstrated with the additional data that was not presented to networks during the training and internal testing and validation procedures. The obtained classification model is in accordance with results of regression models, and can be used as additional tool for the assessment of differences between the formulations.

Based on the overall results that were obtained and presented, it can be concluded that ML algorithms can provide significant aid in understanding tableting properties of co-processed excipients. By developing neural network models, it was possible to successfully identify and compare the influence of several input parameters (five or eleven) on the studied outputs. Compared to the conventional direct analysis of the data or more sophisticated multivariate analysis, where each studied output needs to be analyzed separately, neural networks can simultaneously model multiple outputs. Conventional modeling techniques are usually restrictive in terms of regression analysis of solely numerical data, whereby neural network models of regression type can be developed using categorical variables as well. These are the main benefits of ANNs since significant saving in resources and facilitated development of new products, including multicriteria optimization, can be provided.

## Figures and Tables

**Table 1:** Summary of composition, compression load and excipient state used to prepare tablets (abbreviations: API—active pharmaceutical ingredient, CS—co-processed, PM—physical mixture).

Datasets	API (%)	Compritol^®^ (%)	Precirol^®^ (%)	Lactose Monohydrate (%)	Compression Load (kg)	State of Excipients
Training, testing, and validation data	0	0	0	100	500	PM
		10	0	90		
		0	10	90		
		15	0	85		
		0	15	85		
	25	11.25	0	63.75		
	50	7.50	0	42.50		
	25	0	11.25	63.75		
	50	0	7.50	42.50		
	0	15	0	85		CS
		0	15	85		
	25	11.25	0	63.75		
	50	7.5	0	42.50		
	25	0	11.25	63.75		
	50	0	7.5	42.50		
	0	0	0	100	100	PM
		10	0	90		
		0	10	90		
		15	0	85		
		0	15	85		
	25	11.25	0	63.75		
	50	7.5	0	42.50		
	25	0	11.25	63.75		
	50	0	7.5	42.50		
	0	15	0	85		CS
		0	15	85		
	25	11.25	0	63.75		
	50	7.5	0	42.50		
	25	0	11.25	63.75		
	50	0	7.5	42.50		
External validation dataset	25	11.25	0	63.75	250	PM
		0	11.25	63.75		
		11.25	0	63.75		CS
		0	11.25	63.75		
	50	7.5	0	42.50		PM
		0	7.5	42.50		
		7.5	0	42.50		CS
		0	7.5	42.50		

**Table 2:** Predictions of neuron position for the external validation data (abbreviations: DW—detachment work, EF—ejection force, EJW—ejection work, ER—elastic recovery, NWC—net work of compression, TS—tensile strength, TWC—total work of compression).

API (%)	Compritol^®^ (%)	Precirol^®^ (%)	State	TS (MPa)	TWC (Nm)	NWC (Nm)	ER (%)	DW (Nm)	EJW (Nm)	EF (N)	Neuron Position
25	11.25	0	PM	0.64	1.10	0.93	19.3	0.14	0.58	123	(2,1)
25	0	11.25	PM	0.57	0.81	0.63	21.5	0.09	0.01	30	(2,1)
25	11.25	0	CS	1.05	0.86	0.69	22.5	0.10	0.01	35	(2,2)
25	0	11.25	CS	0.53	0.68	0.51	21.6	0.18	0.03	54	(2,2)
50	7.5	0	PM	0.51	4.05	3.89	17.5	0.12	8.00	692	(2,1)
50	0	7.5	PM	0.30	0.83	0.64	20.9	0.10	0.01	36	(2,1)
50	7.5	0	CS	0.70	0.85	0.68	21.6	0.25	0.01	50	(2,2)
50	0	7.5	CS	0.00	0.76	0.58	21.0	0.30	0.19	190	(2,2)

**Table 3:** The optimal multilayer perceptron (MLP) and radial basis function (RBF) networks developed for single variable outputs (abbreviations: BFGS—Broyden–Fletcher–Goldfarb–Shanno, DW—detachment work, EF—ejection force, EJW—ejection work, ER—elastic recovery, Exp—exponential, NWC—net work of compression, TS—tensile strength, TWC—total work of compression.

Target Output	Optimal Neural Network	Correlation Coefficients				Training Algorithm	Activation Functions	
		Training Data	Test Data	Validation Data	External Validation Data		Hidden Layer	Output Layer
TS	MLP 6-10-1	0.9958	0.9943	0.9994	0.6978	BFGS 98	Exp	Exp
	RBF 6-16-1	0.9685	0.9830	0.9852	0.9218	RBF	Gaussian	Identity
TWC	MLP 6-3-1	0.8920	0.9932	0.9934	0.9996	BFGS 146	Logistic	Exp
	RBF 6-18-1	0.8628	0.9089	0.8553	0.9890	RBF	Gaussian	Identity
NWC	MLP 6-7-1	0.8772	0.9973	0.9944	0.9998	BFGS 177	Logistic	Identity
	RBF 6-16-1	0.8570	0.7915	0.8095	0.9736	RBF	Gaussian	Identity
ER	MLP 6-11-1	0.9786	0.9699	0.9557	0.9554	BFGS 49	Logistic	Tanh
	RBF 6-13-1	0.9114	0.9462	0.9354	0.9448	RBF	Gaussian	Identity
DW	MLP 6-4-1	0.9839	0.9951	0.9736	0.9720	BFGS 107	Exp	Exp
	RBF 6-15-1	0.9534	0.9585	0.8769	0.9379	RBF	Gaussian	Identity
EJW	MLP 6-5-1	0.9151	−0.1924	0.8487	0.9997	BFGS 6	Exp	Logistic
	RBF 6-17-1	0.9531	0.2711	0.8236	0.9715	RBF	Gaussian	Identity
EF	MLP 6-5-1	0.9994	0.9805	0.9772	0.9945	BFGS 147	Tanh	Logistic
	RBF 6-17-1	0.9566	0.6982	0.6248	0.9726	RBF	Gaussian	Identity

**Table 4:** Properties of MLP 12-11-1 network developed for prediction of tablets’ TS. Abbreviations: BFGS—Broyden–Fletcher–Goldfarb–Shanno, TS—tensile strength.

Correlation Coefficients				Errors			Training Algorithm	Activation Functions	
Training Data	Test Data	Validation Data	External Validation Data	Training Data	Test Data	Validation Data		Hidden Layer	Output Layer
0.9999	0.9999	0.9999	0.9263	0.00002	0.00005	0.00006	BFGS 140	Logistic	Identity

**Table 5:** The optimal MLP and RBF networks developed for multiple variable outputs. Abbreviations: BFGS—Broyden–Fletcher–Goldfarb–Shanno, MLP—multilayer perceptron, RBF—radial basis function).

Artificial Neural Networks	Correlation Coefficients				Training Algorithm	Activation Functions	
	Training Data	Test Data	Validation Data	External Validation Data		Hidden Layer	Output Layer
MLP 6-11-7	0.9538	0.8857	0.9328	0.9048	BFGS 151	Logistic	Tanh
MLP 6-11-7	0.9553	0.8716	0.9416	0.9416	BFGS 215	Exponential	Tanh
MLP 6-6-7	0.9451	0.8510	0.9163	0.8572	BFGS 158	Logistic	Exponential
RBF 6-14-7	0.9199	0.6477	0.8200	0.8915	RBF	Gaussian	Identity
RBF 6-16-7	0.9162	0.7197	0.8746	0.9111	RBF	Gaussian	Identity

## Data Availability

The data presented in this study are available on request from the corresponding author.

## Abbreviations

Active pharmaceutical ingredient (API), artificial neural networks (ANN), Broyden–Fletcher–Goldfarb–Shanno (BFGS), co-processing (CP), co-processed excipient (CPE), DW (detachment work), EF (ejection force), EJW (ejection work), ER (elastic recovery), Kohonen network (KN), machine learning (ML), multilayer perceptron (MLP) NWC (net work of compression), physical mixture (PM), radial basis function (RBF), response surface methodology (RSM), root mean squared error (RMSE), self-organizing feature map (SOFM), sum of squares (SOS), tensile strength (TS), total work of compression (TWC).
